# Digitize-HCD: A dataset for digitization of handwritten circuit diagrams

**DOI:** 10.1016/j.dib.2025.111315

**Published:** 2025-01-17

**Authors:** Nadim Ahmed, Mirza Fuad Adnan, Ahmad Shafiullah, Hayder Jahan Parash, Md. Saifur Rahman, Irfan Chowdhury Akib, Golam Sarowar

**Affiliations:** a Department of Electrical and Electronic Engineering, Islamic University of Technology, Gazipur 1704, Bangladesh; b Department of Electrical, Electronic and Communication Engineering, Military Institute of Science and Technology, Dhaka, Bangladesh

**Keywords:** Computer vision, Diagram digitization, Handwritten circuit diagram, Circuit component detection, Text recognition

## Abstract

Handwritten and legacy engineering diagrams are still commonly used in many industries and academic settings, but the lack of digitization limits their utility in modern workflows. While significant effort has been made to digitize handwritten content in other engineering domains, the digitization of handwritten circuit diagrams remains underexplored. Automating this process would enable the development of tools capable of converting handwritten circuit diagrams into machine-readable formats that can be instantly interpreted by circuit simulation software. This will offer significant benefits to both students and industry professionals. However, the lack of publicly available datasets focused on the digitization of handwritten circuit diagrams has slowed progress in this area. To address this gap, we developed the Digitize - Handwritten Circuit Diagram (HCD) Dataset, a comprehensive collection of 1,277 handwritten circuit diagrams contributed by 176 volunteers. The dataset includes detailed annotations for multiple aspects of handwritten circuit diagrams, such as component symbols, text labels, and port locations. It contains 18,602 annotated instances across 17 distinct classes of circuit component symbols and 11,936 annotated text labels associated with these components. For the preparation of ground-truth data for component port localization, we developed an annotation tool, which is publicly available for reuse. The Digitize-HCD dataset has the potential to accelerate research on digitization of handwritten circuit diagrams and contribute to the development of advanced end-to-end tools capable of transforming these diagrams into machine-readable formats.

Specifications Table

**Table:** 

Subject	Computer Vision and Pattern Recognition
Specific subject area	Digitization of Handwritten Engineering Diagrams
Type of data	∗.jpg (images of handwritten circuit diagrams and extracted component images), ∗.json (annotations for component symbols and text labels), ∗.png (ground-truth heatmaps for component port location), ∗.txt (text files for port coordinate data)
Data collection	To prepare the dataset, a total of 1,277 circuit diagrams were drawn on paper by 176 participants. The students of Department of Electrical and Electronic Engineering, Islamic University of Technology, Bangladesh volunteered to draw necessary circuit diagrams for the datasets. All images were drawn on white A4 paper, using pen or pencil. After collecting the drawn circuit diagrams, images were captured using scanner, with a resolution of 600 dpi. The scanned images were resized and cropped to only the diagram, removing the unnecessary white backgrounds.
Data source location	Institution: Department of Electrical and Electronic Engineering, Islamic University of Technology Location: Boardbazar, Gazipur-1704, Dhaka, Bangladesh Latitude and longitude: 23° 56′ 53.1" N, 90° 22′ 45.4" E
Data accessibility	Repository name: Mendeley Data Data identification number: 10.17632/rngcz5wtv8.1 Direct URL to data: https://data.mendeley.com/datasets/rngcz5wtv8/1
Related research article	

## 1 Value of the Data

• This dataset serves as a comprehensive resource for research in the field of digitization of engineering diagrams, specifically focusing on the underexplored domain of handwritten electrical and electronic circuit diagram digitization. The dataset can serve as a valuable benchmark for researchers working on similar tasks in the field of handwritten diagram digitization.
• The dataset includes detailed annotations for multiple aspects of handwritten circuit diagrams, such as component symbols, text labels, and port locations. These annotations support the development of end-to-end digitization tools capable of converting handwritten circuit diagrams into digital, machine-readable formats.
• The circuit diagrams in the dataset contain a wide variety of handwriting styles, contributed by 176 volunteer drafters. This diversity enhances robustness of the dataset and ensures its suitability for real-world applications, where variability in handwriting is a common challenge.
• Potential applications of this dataset include the development of tools that can capture images of hand-drawn circuit diagrams and instantly simulate their behavior. Such tools would significantly improve design accessibility and efficiency for a broader audience, including students, educators, and professionals.
• The availability of such a dataset can accelerate the development of Electronic Design Automation (EDA) tools capable of integrating handwritten diagram recognition with simulation and design processes, bridging the gap between traditional drawing methods and modern engineering workflows.

## 2 Background

A significant portion of engineering diagrams currently in use are still in handwritten or legacy format [1]. Despite advances in digital tools, many industries still rely on these traditional, manually created diagrams, which often require digitization efforts to integrate them into modern workflows. Research across various engineering fields has focused on digitizing handwritten and legacy diagrams for a considerable period, with significant efforts made in understanding and analyzing these graphical representations [2]. This Includes areas such as mechanical drawings [3], flowcharts [4], structural designs [5], process flow diagrams [6], piping and instrumentation diagrams (P&IDs) [7], chemical structures [8], etc. However, certain applications within this domain have received comparatively less attention. One such under-explored area is digitization of handwritten circuit diagrams.

A circuit diagram is a visual representation of an electrical or electronic circuit, showing the components and their connections in a system using standardized symbols [9]. Simulation of circuit diagrams is crucial for analyzing, designing, and verifying electrical circuits. Currently, a good number of simulation tools are available, providing robust platforms for simulation of circuits. They offer a range of features from basic circuit analysis to advanced simulations involving complex electronic systems. Despite the capabilities of these platforms, the process of manually digitizing and preparing circuit diagrams for simulation can be time-consuming and prone to errors, especially when dealing with handwritten sketches or legacy diagrams. The development of a tool that can capture images of hand-drawn circuit diagrams and then instantly and accurately simulate the circuit would greatly enhance design accessibility and efficiency. Such a tool would cater to a wider audience, including students, educators, and professionals who may not have extensive experience with traditional circuit design software. In educational settings, the ability to quickly simulate circuits from images could revolutionize the learning experience, enabling students to experiment freely with circuit designs and immediately see the results of their work. For professionals, the time saved in converting sketches to simulation-ready formats could accelerate the design process, allowing for more time to be spent on innovation, optimization, and testing.

However, current research on digitization of handwritten circuit diagrams is still in its early stages and remains relatively limited in scope, primarily due to the scarcity of publicly available datasets in this domain. Existing datasets are often constrained by size, diversity, and scope [[10], [11], [12]]. Firstly, these datasets are developed under controlled conditions and lack a wide range of handwriting styles. Furthermore, many of these datasets do not contain enough data samples to effectively train and evaluate robust models. Lastly, none of the currently existing datasets provide annotations for critical aspects such as localization or connectivity detection, focusing instead on specific tasks like classification or object detection. To address this gap, the Digitize-HCD dataset was developed with an aim of facilitating further research on digitization of handwritten circuit diagrams. This dataset includes detailed annotations for multiple aspects of handwritten circuit diagrams, such as component symbols, text labels, and port locations. The dataset features contributions from 176 volunteer drafters, offering a wide range of handwriting styles. This diversity enhances robustness of the dataset and ensures its suitability for real-world applications, where variability in handwriting is a common challenge. Digitize-HCD dataset can potentially contribute to the development of advanced end-to-end digitization tools capable of transforming handwritten circuit diagrams into accurate, machine-readable digital formats.

## 3 Data Description

**Fig. 1:**
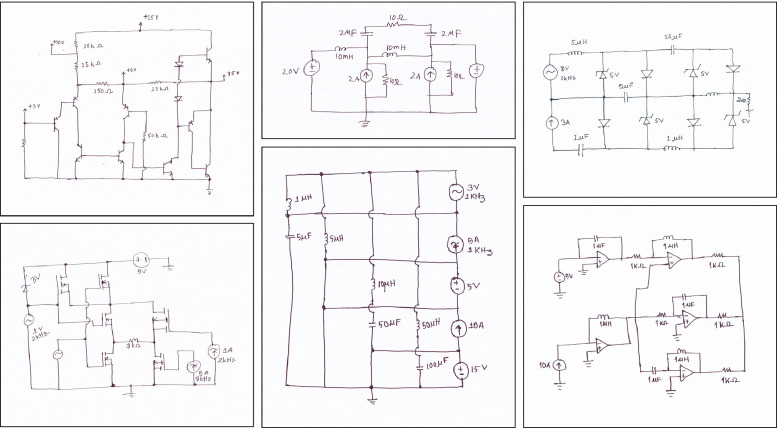
Sample images from collected handwritten circuit diagrams.

The Digitize-HCD dataset is derived from a collection of 1,277 images of handwritten circuit diagrams. All diagrams were drawn on white A4 paper, using pen or pencil. From the collected drawings of circuit diagrams, images were captured using scanners, with a resolution of 600 dpi. The finalized diagram images were stored in JPG format. Fig. 1 shows sample handwritten diagrams produced from this process. Collected diagram images cover 16 different categories of components in total. The annotations extracted from these images can be classified into three categories: (i) component symbol detection dataset, (ii) component text label detection and recognition dataset, and (iii) component port localization dataset. The Digitize-HCD dataset is publicly available in Mendeley Data [13].

### 3.1 Component symbol detection dataset

**Fig. 2:**
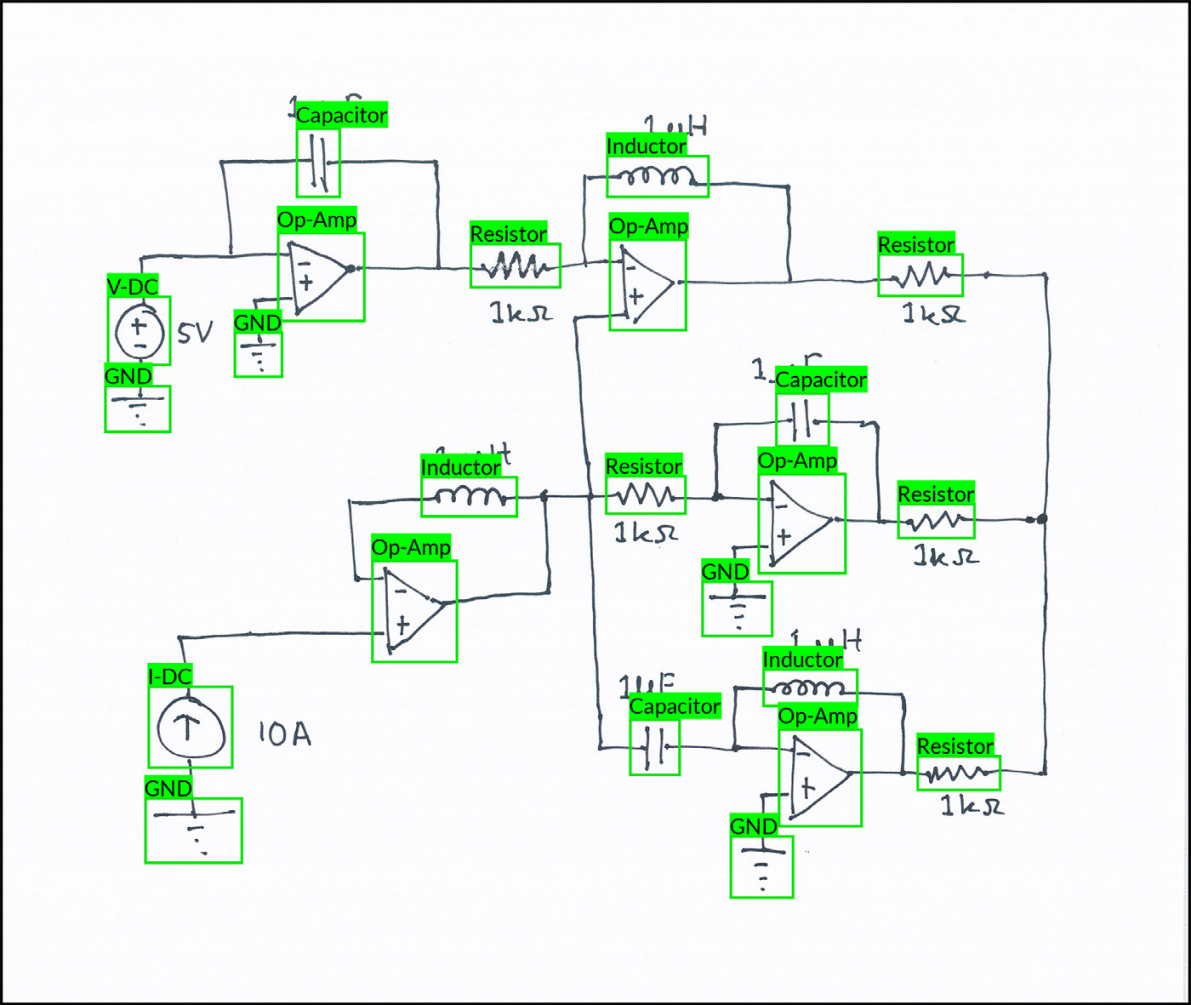
Sample diagram image containing annotations for component symbol detection.

**Fig. 3:**
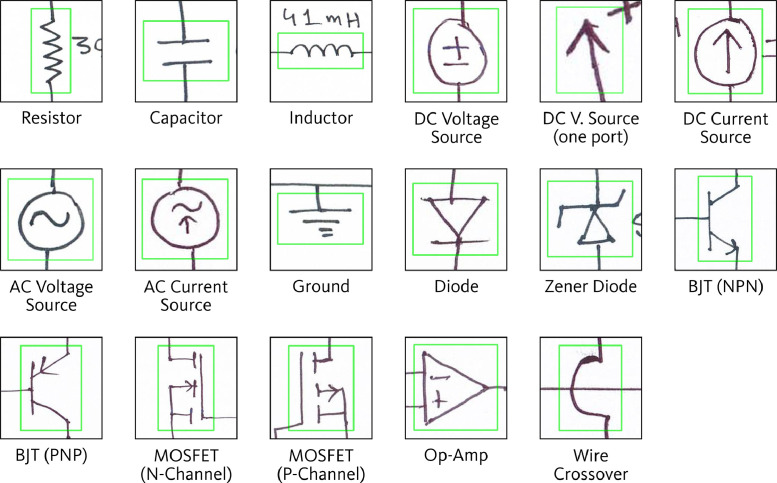
Representative sample from each of the 17 symbol classes in the component symbol detection dataset.

**Fig. 4:**
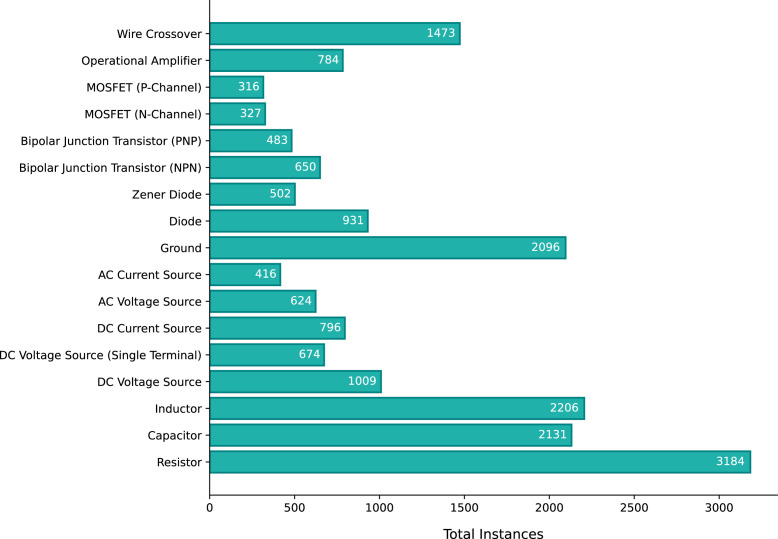
Distribution of instances across different component symbol classes.

All component symbols in the collected set of 1,277 images were manually annotated, including both classification and localization of each symbol. For localizing the components, Axis-Aligned Bounding Boxes (AABBs) were used. Fig. 2 shows a sample diagram image containing annotations for component symbol detection. The annotation process resulted in a total of 18,602 annotations spanning 17 symbol classes. Sixteen of these classes correspond to various circuit components, while the remaining class serves as an auxiliary category, designated for wire crossovers. Wire crossover represents two wires that are crossing paths in the diagram but are not forming an electrical connection at that crossing point. Fig. 3 shows representative sample from each of the 17 symbol classes in the dataset. Fig. 4 presents the distribution of instances across the different classes.

The annotation data is stored in a JSON file using an annotation format adapted from the standardized format and conventions used by the COCO (Common Objects in Context) dataset [14]. This annotation format includes three main sections, i.e., *“categories”, “images”*, and *“annotations”*. Details of each section are as follows:

• ***“categories”*:** The categories section defines the types of circuit components represented within the dataset. Each component type is assigned a unique numerical identifier (*“id”*) and a descriptive label (*“name”*).
• ***“images”*:** This section contains metadata about the individual images of handwritten circuit diagrams. Each image is assigned a unique identifier (*“id”*), a reference to the filename (*“file_name”*), and its dimensions in pixels (*“height”* and *“width”*).
• ***“annotations”*:** The *“annotations”* section contains detailed information about each identified component symbol in the images. Each annotation links to a specific image and provides information about the location and properties of the component symbol within that image. The parameters for each annotation include:
  ○ ***“id”*:** A unique identifier for each annotation.
  ○ ***“image_id”:*** The identifier of the image where the component is located, linking the annotation to a specific image from the *“images”* section.
  ○ ***“category_id”:*** Refers to the corresponding component symbol class, as defined in the “categories” section.
  ○ ***“bbox”:*** The bounding box that encloses the component. It is represented by an array of four values, with the format [x,y,width,height]. x and y represent the coordinates of the top-left corner of the bounding box. width and height represent the dimensions of the bounding box in pixels.
  ○ ***“area”:*** The area of the bounding box, representing the number of pixels the component occupies.
  ○ ***“segmentation”:*** A list of coordinates defining the polygonal outline of the component. As segmentation data was not extracted for the dataset, this field is empty.
  ○ ***“iscrowd”:*** A boolean value (0 or 1) that indicates whether the component is part of a cluster or crowd of objects.

### 3.2 Component text label detection and recognition dataset

**Fig. 5:**
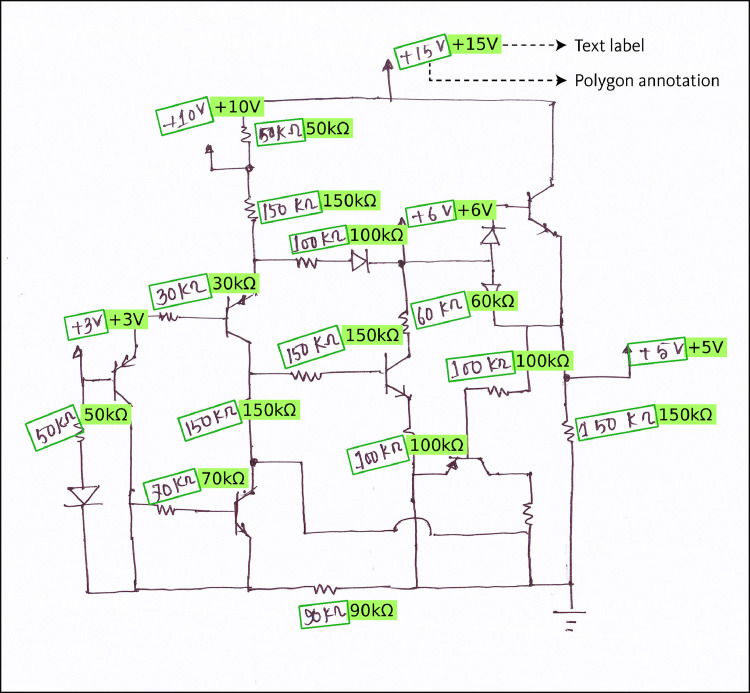
A sample image of annotated diagram image from the text detection and recognition dataset.

From the collected images of circuit diagrams, the text labels of component symbols were annotated. This annotation data includes detailed annotations for these text regions, with each text label localized using polygonal annotations that define the exact boundaries. Additionally, each annotated polygon includes the corresponding text label bounded by the region, providing both the location and the corresponding textual content. Fig. 5 shows a sample annotated image from the developed dataset. From the collection of 1,277 circuit diagram images, 11,936 text strings were localized using polygon annotations. These annotated text strings comprise a total of 44,443 characters. The annotated texts within the dataset provide detailed descriptions of key electrical properties of components. Specifically, they include annotations for properties such as resistance, capacitance, inductance, voltage or current values for sources, etc. The character set includes numbers, few upper and lowercase English alphabets, mathematical operators, and the Greek letters Ω and μ.

**Fig. 6:**
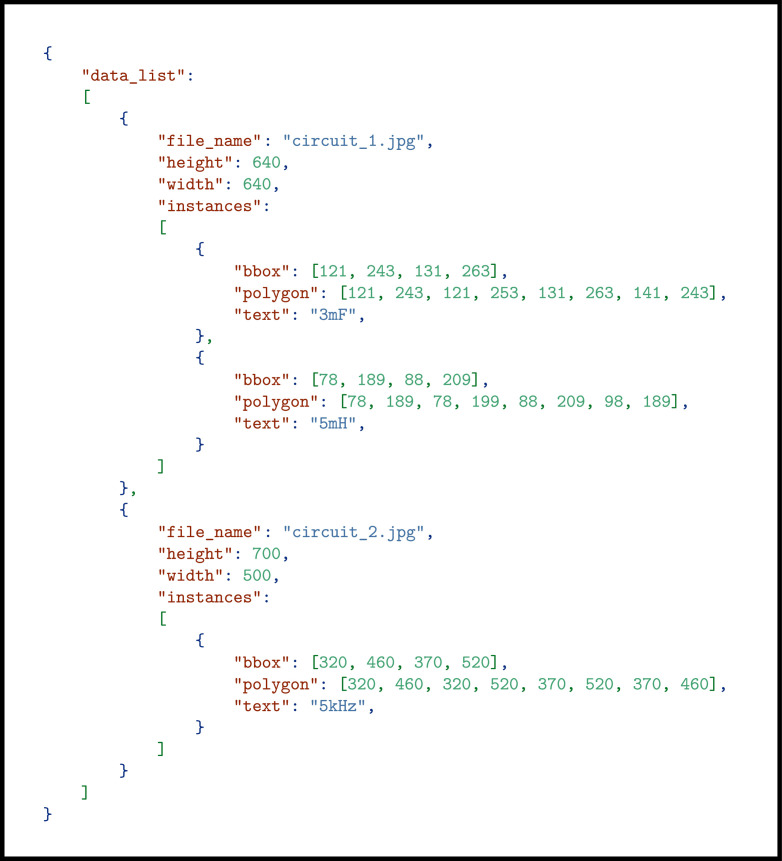
A sample JSON file consistent with the annotation format used for text label detection and recognition dataset.

To store the data, an annotation format was adopted from the OCRDataset format of MMOCR [15]. In this annotation format, data is stored in a JSON file. Fig. 6 shows an exemplary JSON file consistent with the annotation format used in this dataset. This file primarily consists of a *“data_list”* section, which contains detailed annotations for each target instance within the dataset. Each entry in the *“data_list”* includes:

• ***“file_name”:*** Name of the image file with extension.
• ***“height”:*** The height of the image in pixels.
• ***“width”:*** The width of the image in pixels.
• ***“instances”:*** An array of instances, where each instance represents a text element within the image. Each instance includes:
  ○ ***“polygon”:*** The coordinates of the polygon enclosing the text, formatted as [x1,y1,x2,y2,...].
  ○ ***“bbox”:*** The bounding box coordinates [x1,y1,x2,y2], representing the top-left and bottom-right corners of the bounding box.
  ○ ***“text”:*** Textual content within the polygon.

### 3.3 Component port localization dataset

The component port localization dataset was developed specifically for identifying and localizing ports of components within the circuit diagram. This dataset can help in understanding the connectivity among the components within a circuit. The annotation data from component symbol detection dataset served as the basis for deriving this dataset.

In the component symbol detection dataset, all component symbols in the diagram are annotated using bounding boxes. From each these annotated component symbols, a region surrounding the symbol was cropped to serve as input sample for the component port localization dataset. The cropping process retained the original bounding box of the annotated component symbol within the extracted image. All generated input samples have a fixed resolution of 320 × 320 pixels.

**Fig. 7:**
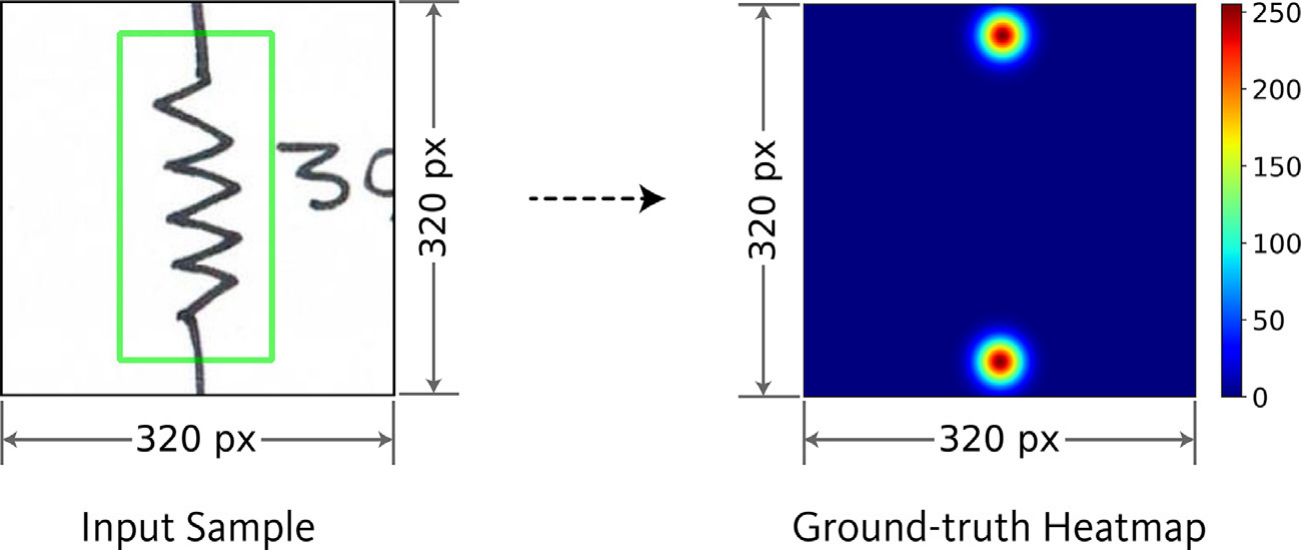
Example of an input sample and its corresponding ground-truth heatmap in component port localization dataset.

For representing the port locations in input samples, probability heatmaps are used as ground-truth. The ground-truth heatmaps have dimensions of 320 × 320 pixels, similar to the input sample. Each pixel in the heatmap is assigned a value ranging from 0 to 255, indicating the likelihood that the pixel contains a component port. In the input sample images, the component port locations were defined as the coordinates where the port wires extend and intersect with the bounding boxes. The likelihood of port presence in the ground-truth heatmap were modeled using two-dimensional Gaussian kernels centered at these port coordinates. The exact XY coordinate values of the port locations were also saved in text files for further reference. Fig. 7 shows an example of an input sample and its corresponding ground-truth heatmap.

**Fig. 8:**
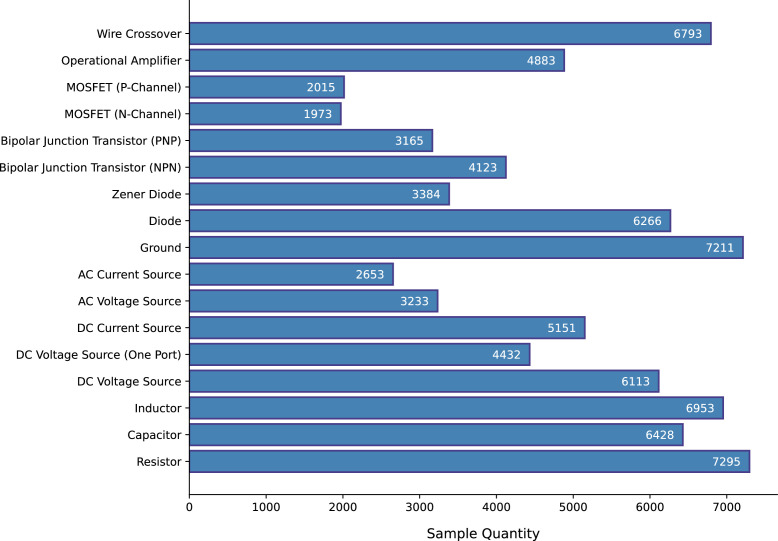
Sample quantities within each class-specific dataset of the port localization dataset.

**Fig. 9:**
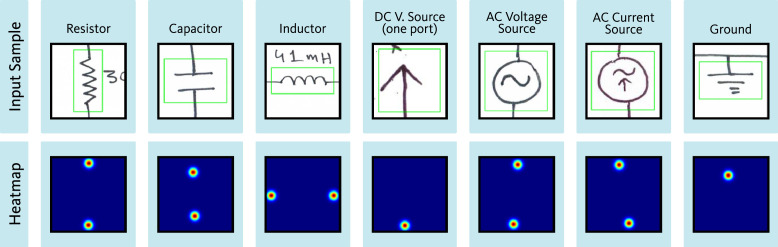
Representative input and the corresponding ground-truth heatmap for symbol categories that require a single heatmap as ground-truth.

**Fig. 10:**
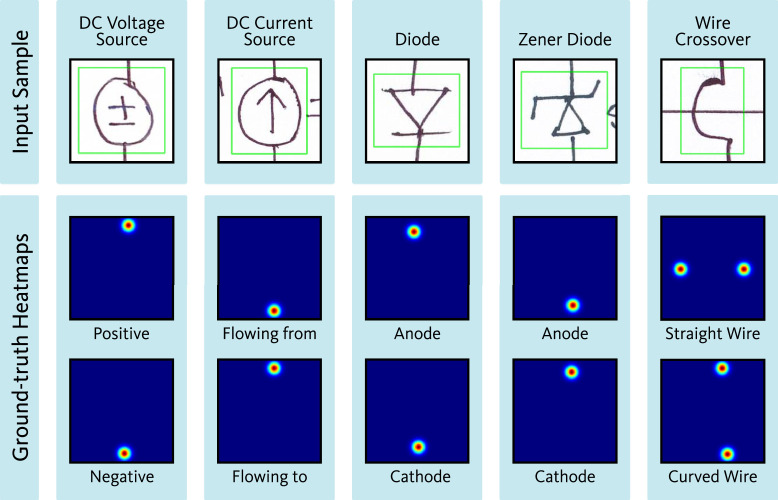
Representative input along with their corresponding ground-truth heatmaps for symbol categories that require two heatmaps as ground-truth.

**Fig. 11:**
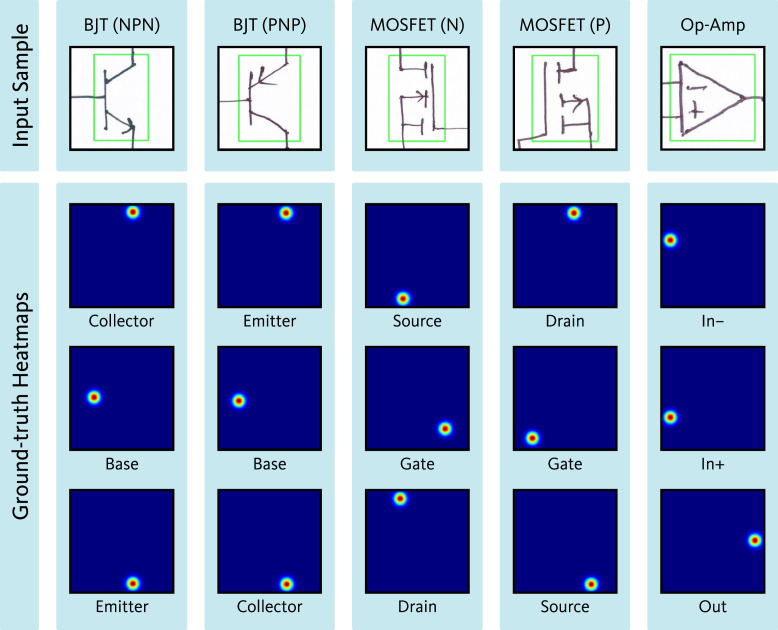
Representative input along with their corresponding ground-truth heatmaps for symbol categories that require three heatmaps as ground-truth.

For each of the 17 symbol classes (16 component classes and one auxiliary class), a separate dataset was prepared. Fig. 8 presents the sample quantities within each class-specific dataset of the port localization dataset. Among the 16 component classes, certain components possess only one port. Additionally, for some two-port components, such as resistors, inductors, AC voltage sources, etc., identifying the orientation of the ports is not critical. For both cases, a single ground-truth heatmap was generated, in which all port locations were represented using distinct 2D Gaussian kernels. Fig. 9 presents representative input and the corresponding ground-truth heatmap for symbol categories that require a single heatmap as ground-truth. In contrast, some other component classes require specific orientation identification for each port, such as MOSFET. For MOSFET, all three ports, i.e., drain, source, and gate, must be identified individually. For components such as this, individual ground-truth heatmaps were generated for each port. Each of these ground-truth heatmaps represents the location of one of the ports using a 2D Gaussian. For the auxiliary class representing wire crossovers, two separate heatmaps were generated. Each heatmap contains the port locations for one of the two overlapping wires. Fig. 10, Fig. 11 show representative input along with their corresponding ground-truth heatmaps for symbol categories that require two and three heatmaps, respectively, as ground-truth.

### 3.4 Dataset directory structure

**Fig. 12:**
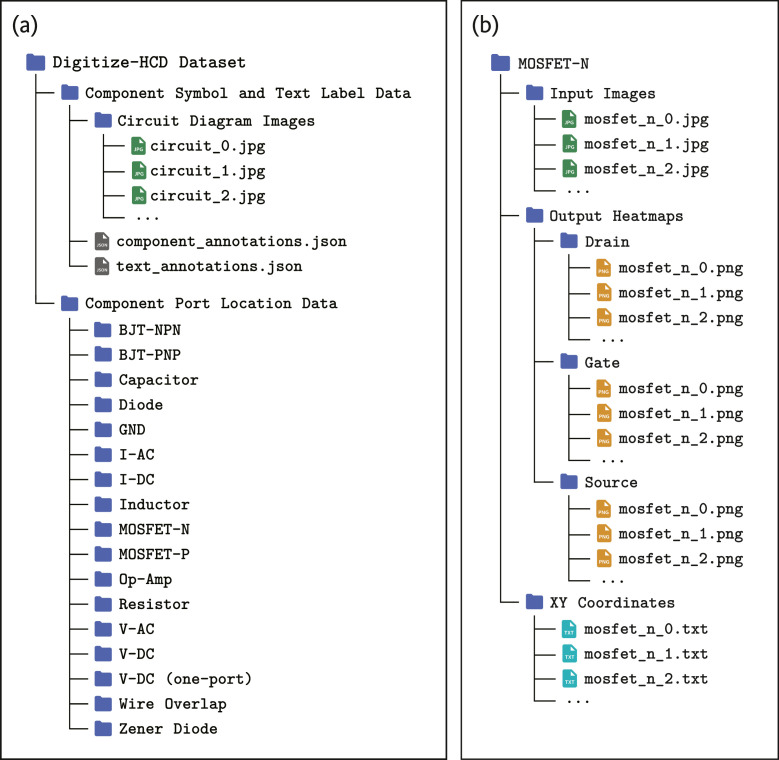
Directory structure of Digitize-HCD dataset. **(a)** Main directory of the dataset containing two subdirectories “Component Symbol and Text Label Data” and “Component Port Location Data”. **(b)** Example of the directory structure for port location data specific to a particular component (N-channel MOSFET) within the subdirectory “Component Port Location Data”.

Digitize-HCD dataset is organized into two main subdirectories: *“Component Symbol and Text Label Data”* and *“Component Port Location Data”*. Fig. 12(a) provides a visual representation of overall directory structure of the dataset.

The subdirectory *“Component Symbol and Text Label Data”* contains a folder and two JSON files. The folder named *“Circuit Diagram Images”* contains the collected 1,277 JPG images of handwritten circuit diagrams. The two JSON files in this directory are, *“component_annotations.json”* and *“text_annotations.json”*. The file *“component_annotations.json”* contains annotations for component symbol detection and *“text_annotations.json”* file includes the annotations for text label detection and recognition.

The second subdirectory, *“Component Port Location Data”*, contains the port localization datasets developed for each of the component symbol categories. The directory contains 17 different folders, each folder dedicated to a specific component symbol class. Fig. 12(b) illustrates an example of the data organization within each folder. As shown in Fig. 12(b), *“MOSFET-N”* contains the following subdirectories:

• *“Input Images”:* This folder stores the original input samples of 320 × 320 pixels extracted for the specific component class (e.g., MOSFET-N).
• *“Output Heatmaps”:* This folder holds the ground-truth heatmaps for each input sample. For example, for a MOSFET (N-channel), heatmaps are generated for its three distinct terminals: Drain, Gate, and Source. Location of each port is represented as a separate probability heatmap stored in its respective subfolder (e.g., *“Drain”, “Gate”* and *“Source”*).
• *“XY Coordinates”*: The exact XY coordinate values of the port locations were also stored in text files for further reference.

## 4 Experimental Design, Materials and Methods

This section outlines the methodology employed in developing the Digitize-HCD dataset, including the collection of handwritten circuit diagrams and the annotation processes for the three datasets: (i) the component symbol detection dataset, (ii) the component text label detection and recognition dataset, and (iii) the component port localization dataset. Subsections below provide a detailed description of each stage of the process.

### 4.1 Collection procedure for handwritten diagrams

To prepare these datasets, a total of 1,277 circuit diagrams were drawn on paper by 176 participants. The students of Department of Electrical and Electronic Engineering, Islamic University of Technology, Bangladesh volunteered to draw necessary circuit diagrams for the datasets. The circuit diagrams cover 16 different classes of components in total. Corresponding text labels of all components were included in the diagrams. All images were drawn on white A4 paper, using pen or pencil.

Two different approaches were taken for drawing the diagrams. In one of the approaches, diagrams were drawn by volunteers by following given templates of circuit diagrams. Volunteers recreated the given templates by drawing them on paper. In another approach, diagrams were drawn spontaneously without following any template diagrams. Employing these two approaches ensured less class imbalance at the same time diversity of the dataset. To ensure the quality of the datasets, some poorly drawn circuit diagrams were discarded. In these diagrams, components, associated text or their connections were not recognizable by humans.

After collecting the drawn circuit diagrams, images were captured using scanner, with a resolution of 600 dpi. The scanned images were resized and cropped to only the diagram, removing the unnecessary white backgrounds. The finalized diagram image sizes range between 1053 × 797 pixels and 2869 × 3208 pixels and are stored in JPG format.

### 4.2 Component symbol and text label annotation

All components in the collected 1,277 images were manually annotated for classification and localization. The datasets were annotated using Roboflow [16], an open-source platform for managing and labeling computer vision datasets. The dataset spans 17 classes of circuit diagram symbols. Sixteen of these classes correspond to various circuit components, while the remaining class serves as an auxiliary category, designated for wire crossovers. Crossover represents two wires that are crossing paths in the diagram but are not forming an electrical connection at that crossing point. The purpose of annotating crossover instances is to assist the digitization framework in accurately detecting connections between the different components in the circuit diagram. For localizing the components, Axis-Aligned Bounding Boxes (AABBs) were used. The AABBs were represented as tuples (x,y,width,height) in the dataset. Here, (x,y) are the coordinates of the top-left corner of the bounding box, and width and height define the dimensions of the box. The AABBs were selected for component symbol annotation as most state-of-the-art object detection models are specifically designed and optimized to work with AABBs, making them the standard for training and evaluation. Furthermore, the majority of component symbols in the dataset exhibit alignment along a grid or dominant axis, which is a characteristic feature of structured circuit diagrams.

For annotating the textual contents within the collected circuit diagram images, a two-stage annotation process was employed. First, text strings in the images were localized by manually annotating their bounding regions using polygon annotations. Polygon annotations were selected rather than rectangular bounding boxes, as many of the text labels in the diagrams are slightly rotated relative to the conventional horizontal reading direction. Polygon annotations allowed precise localization of the boundaries of these rotated text regions. In the second stage, the textual content within each annotated polygon region was manually transcribed for text recognition. For polygon annotation, Roboflow annotation platform was used. However, for transcribing the textual contents, a manual approach was taken using python scripts.

### 4.3 Extracting input samples for component port localization dataset

**Fig. 13:**
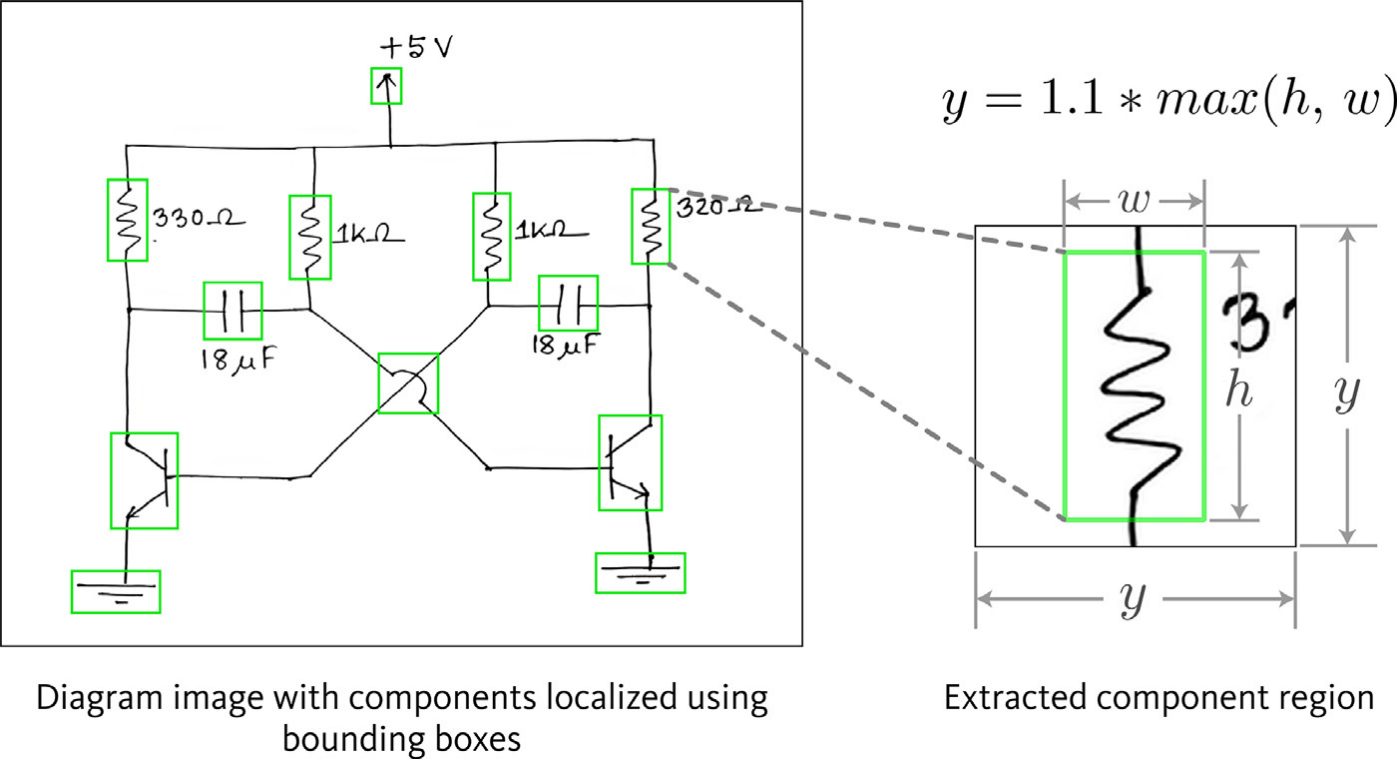
Extraction of input samples for component port localization dataset.

As mentioned previously, the component symbol detection dataset was utilized to prepare the input samples for port localization dataset. Based on the annotation data from component detection dataset, bounding boxes were drawn surrounding the component symbols in diagram images. Subsequently, from each component in the diagram image, a region surrounding the component was cropped to be used as an input sample for the port localization dataset. The procedure for cropping the input samples is illustrated in Fig. 13. Suppose the width and height of the bounding box of the component are w and h, respectively. First, the maximum value between the w and h was determined. Then, the cropping was performed centering the bounding box of the component, with the length of each side of the cropped region being equal to 1.1×max(h,w). This additional 10% length on each side served as padding. Finally, the cropped region was resized to 320 × 320 pixels to be used as the input sample for the port localization dataset.

Several data augmentation steps were randomly applied within specified ranges to the component symbol detection dataset prior to extracting input samples for the port localization dataset. These augmentations included flipping (horizontal, vertical), rotation (both 90° rotations and within a range of -15° to +15°), shearing (up to 10° in both horizontal and vertical directions), brightness and exposure adjustments (within ±15% and ±10%, respectively), blurring (up to 2px), and noise addition (affecting up to 0.22% of pixels). This augmentation process increased the number of samples per class and introduced data diversity.

Following augmentation, input samples for the port localization dataset were extracted from the augmented component detection images according to the procedure illustrated in Fig. 13. Each input sample was then manually annotated to localize component ports and generate ground-truth data.

### 4.4 Ground-truth generation for port localization dataset

**Fig. 14:**
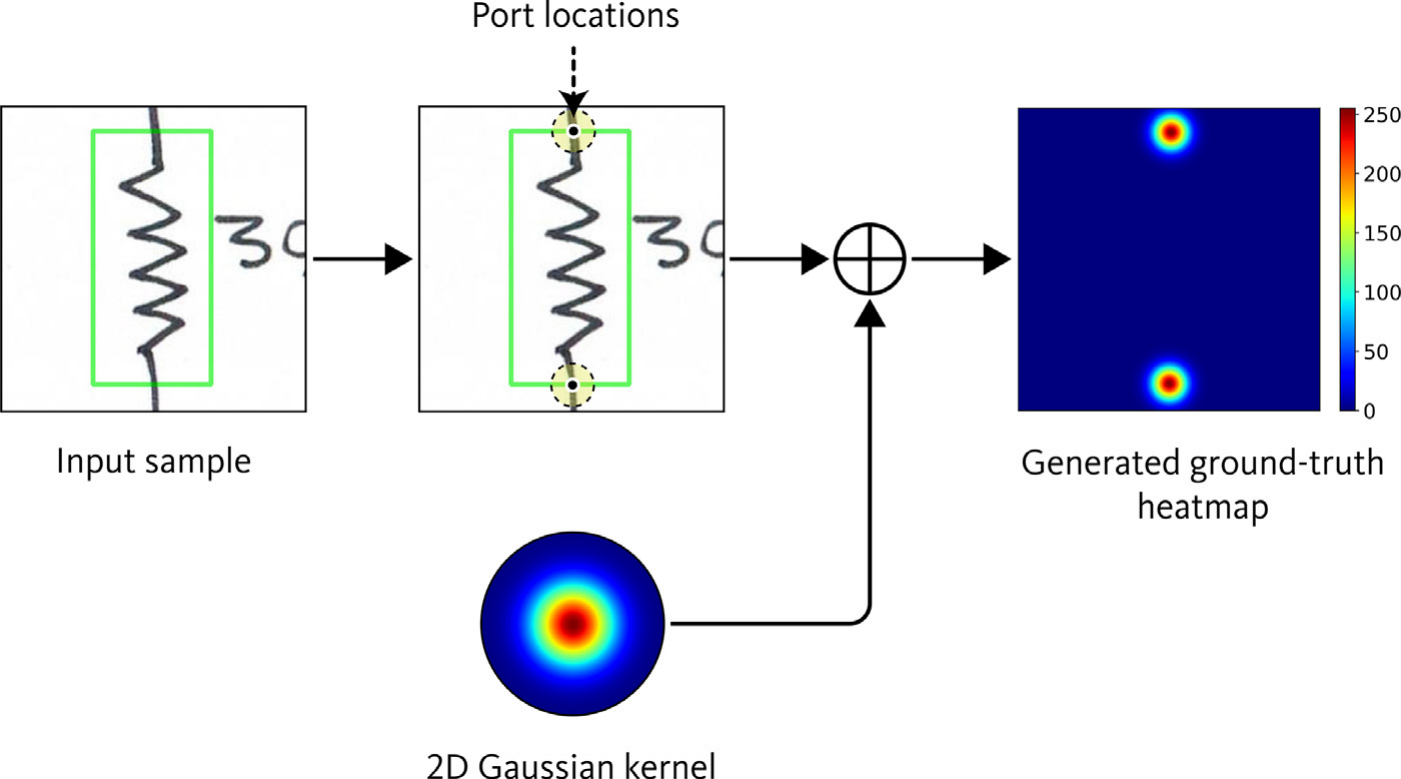
Procedure for generating ground-truth heatmap for port localization dataset.

In the component port localization dataset, ground-truth is represented using a heatmap, which is a grayscale image of the same dimensions as the input sample. In this ground-truth heatmap, each pixel denotes a probability value, ranging from 0 to 255, where 0 indicates the minimum probability and 255 indicates the maximum probability. The procedure for representing the component ports in the ground-truth heatmap is illustrated in Fig. 14. First, locations of component ports were determined by identifying where the port wires extend through and intersect the bounding boxes. The intersection points of these wires with the bounding boxes were designated as the port coordinates. Then, a 2D Gaussian kernel is used to represent the likelihood of the presence of port coordinate in the ground-truth heatmap. The center of the 2D Gaussian kernel is placed at the coordinate of the component port, and the spread of the kernel is controlled by the standard deviation (σ). The value at any point (x,y) on the ground-truth heatmap is determined by the Gaussian function:

(1) $$H\left(x,y\right)=\frac{1}{\sigma \sqrt{2\pi }}\;\text{exp}\left(-\frac{{\left(x-x_{p}\right)}^{2}+{\left(y-y_{p}\right)}^{2}}{2\sigma^{2}}\right)$$

Where, $(x_{p},y_{p})\;$is the coordinate of the port, and σ is the standard deviation. Finally, the generated heatmap was normalized to a range of 0 to 255.

**Fig. 15:**
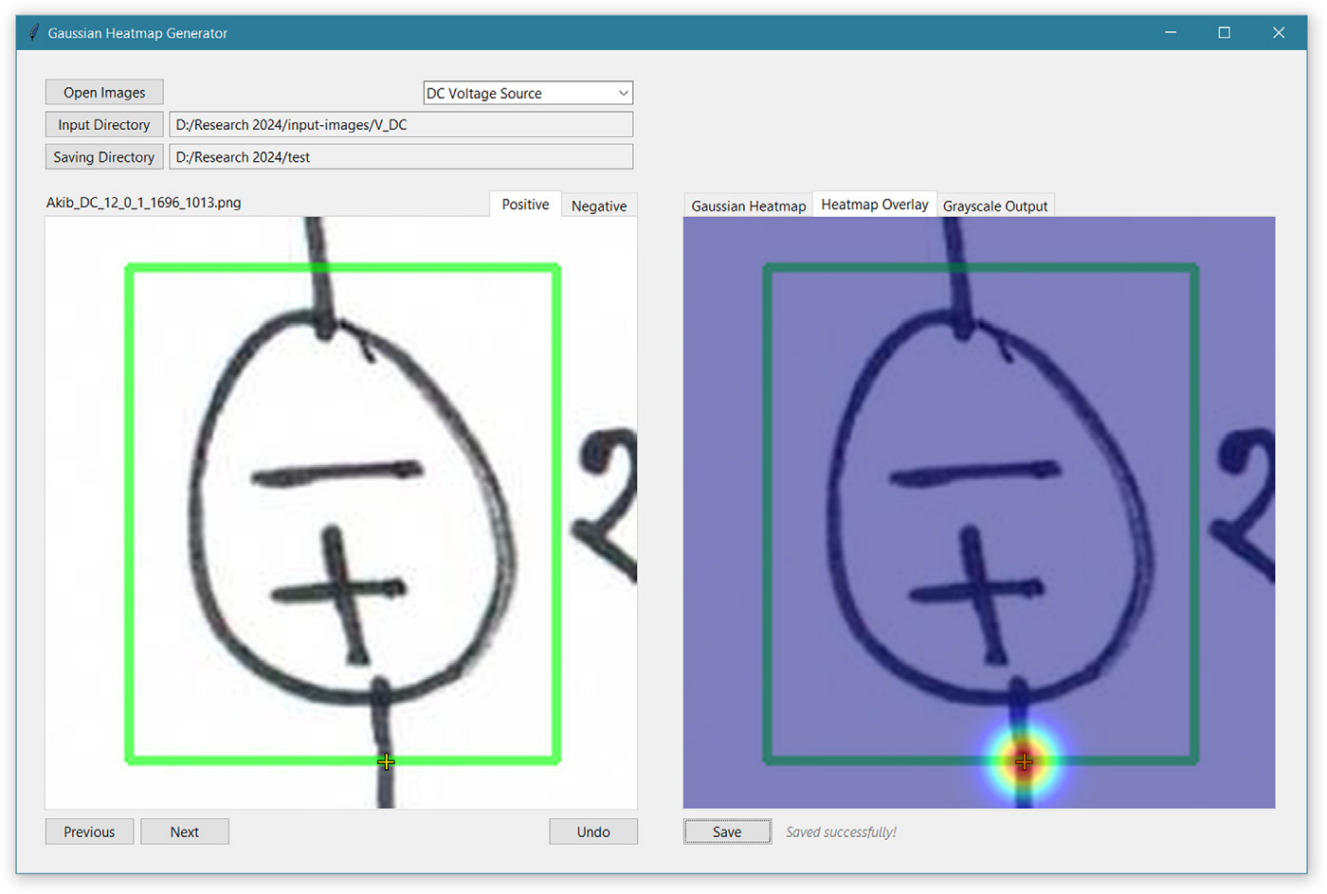
Software developed to generate ground-truth heatmap for port localization dataset.

As part of this research, a custom software was developed to generate ground-truth heatmaps, and it has been made publicly accessible through a GitHub repository [17]. Fig. 15 shows the general inference of the software developed to generate ground-truth heatmap for port localization dataset. In this interface, the left panel contains the input samples of components enclosed within a green bounding box. The port locations are defined as the intersection points where the wire of the component crosses the bounding box. Users can manually click on these intersection points to generate a heatmap with 2D Gaussian kernels centered on these port coordinates. At the top of the interface, a dropdown menu allows users to choose from 17 different component symbols to generate ground-truth heatmap for each type.

## Limitations

The Digitize-HCD Dataset, while comprehensive in many aspects, has several limitations that are important to acknowledge. Firstly, the dataset currently covers only 16 circuit component types. While these are representative of common components, the limited coverage may reduce its applicability to less frequently encountered circuit components. Most component types in the dataset adhere to standardized representations defined by either IEC 60617 or IEEE 315 [18]. However, some component types, such as ‘AC Current Source’ or ‘DC Voltage Source (Single Terminal)’, do not strictly conform to these standards. This is because the dataset was developed in an educational setting, where such symbols are commonly used to teach fundamental concepts of circuit analysis but are less prevalent in standardized industrial documentation.

Additionally, the number of instances per component class is not uniformly distributed, resulting in an imbalance that could influence the performance of machine learning models trained on this data, particularly for classes with fewer samples.

Another limitation stems from the method used to capture the images in the dataset. All images were obtained through a scanning process, which provided high-quality and accurate representations of the handwritten circuit diagrams. However, this approach lacks the variability that might be introduced if images were captured using a camera, such as differences in lighting conditions, angles, or subtle distortions. Incorporating camera-captured images in future versions of the dataset would increase its diversity and enhance its robustness for real-world applications where such variations are often present.

## Ethics Statement

Ethical approval was obtained from the Ethics Approval Committee of Islamic University of Technology, Bangladesh before conducting the study (Reference Number: IUT-EAC-001). Informed consent was obtained from all participants prior to their involvement in the study. Participants were informed about the purpose of the study, their voluntary participation, the confidentiality of their data, and their right to withdraw at any point without consequence. All identifying information was anonymized during dataset preparation to ensure participant confidentiality. The dataset generated from this research is publicly accessible, containing only anonymized contributions of the participants.

## Credit Author Statement

**Nadim Ahmed:** Conceptualization, Methodology, Software, Validation, Formal analysis, Resources, Data Curation, Writing - Original Draft, Writing - Review & Editing, Visualization; **Mirza Fuad Adnan:** Conceptualization, Validation, Resources, Data Curation, Writing - Review & Editing; **Ahmad Shafiullah:** Software, Validation, Resources, Data Curation; **Hayder Jahan Parash:** Validation, Resources, Data Curation; **Md. Saifur Rahman:** Validation, Resources, Data Curation; **Irfan Chowdhury Akib:** Validation, Resources, Data Curation; **Golam Sarowar:** Supervision, Project administration, Writing - Review & Editing.

## Data Availability

Mendeley DataDigitize-HCD: A Dataset for Digitization of Handwritten Circuit Diagrams (Original data).
